# Spatial codistribution of HIV, tuberculosis and malaria in Ethiopia

**DOI:** 10.1136/bmjgh-2021-007599

**Published:** 2022-02-22

**Authors:** Kefyalew Addis Alene, Ahmed Elagali, Dylan D Barth, Susan F Rumisha, Punam Amratia, Daniel J Weiss, Kendalem Asmare Atalell, Andargachew Kumsa Erena, Peter W Gething, Archie C A Clements

**Affiliations:** 1 Geospatial Health and Development, Telethon Kids Institute, Nedlands, Western Australia, Australia; 2 Faculty of Health Sciences, Curtin University, Perth, Western Australia, Australia; 3 University of Western Australia, Perth, Western Australia, Australia; 4 Wesfarmers Centre for Vaccines and Infectious Diseases, Telethon Kids Institute, Nedlands, Western Australia, Australia; 5 National Institute for Medical Research, Dar es Salaam, Tanzania; 6 School of Nursing, College of Medicine and Health Sciences, University of Gondar, Gondar, Ethiopia; 7 National TB Control Program, Ministry of Health, Addis Ababa, Ethiopia

**Keywords:** HIV, tuberculosis, malaria

## Abstract

**Background:**

HIV, tuberculosis (TB) and malaria are the three most important infectious diseases in Ethiopia, and sub-Saharan Africa. Understanding the spatial codistribution of these diseases is critical for designing geographically targeted and integrated disease control programmes. This study investigated the spatial overlap and drivers of HIV, TB and malaria prevalence in Ethiopia.

**Methods:**

HIV, TB and malaria data were obtained from different nationwide prevalence surveys, and geospatial covariates were obtained from publicly available sources. A Bayesian model-based geostatistical framework was applied to each survey leveraging the strength of high-resolution spatial covariates to predict continuous disease-specific prevalence surfaces and their codistribution.

**Results:**

The national prevalence was 1.54% (95% CI 1.40 to 1.70) for HIV, 0.39% (95% CI 0.34 to 0.45) for TB and 1.1% (95%CI 0.95 to 1.32) for malaria. Substantial subnational variation was predicted with the highest HIV prevalence estimated in Gambela (4.52%), Addis Ababa (3.52%) and Dire Dawa (2.67%) regions. TB prevalence was highest in Dire Dawa (0.96%) and Gambela (0.88%), while malaria was highest in Gambela (6.1%) and Benishangul-Gumuz (3.8%). Spatial overlap of their prevalence was observed in some parts of the country, mainly Gambela region. Spatial distribution of the diseases was significantly associated with healthcare access, demographic, and climatic factors.

**Conclusions:**

The national distribution of HIV, TB and malaria was highly focal in Ethiopia, with substantial variation at subnational and local levels. Spatial distribution of the diseases was significantly associated with healthcare access, demographic and climatic factors. Spatial overlap of HIV, TB and malaria prevalence was observed in some parts of the country. Integrated control programmes for these diseases should be targeted to these areas with high levels of co-endemicity.

**Key questions**

**What is already known?**

- HIV, tuberculosis (TB) and malaria are the three most important infectious diseases in Ethiopia.
- The synergy between HIV, TB and malaria infection is strong at an individual level.

**What are the new findings?**

- The distribution of HIV, TB and malaria was highly focal in Ethiopia, with substantial variation at subnational and local levels.
- Spatial overlap of high HIV, TB and malaria prevalence was observed in some parts of the country.
- The spatial distribution of the diseases was associated with healthcare access, demographic and climatic factors.

**What do the new findings imply?**

- Multi-disease control approaches should be emphasised to curb the coexisting three infections, however, relevant interventions should be implemented in areas with lower prevalences.
- Improving healthcare access can reduce the burden of HIV, TB and malaria in Ethiopia.
- Geographically targeted service integration may enhance the efficiency and cost-effectiveness of disease control programmes.

## Background

Infectious diseases are significant contributors to the global burden of death and disability.^1^ HIV, tuberculosis (TB) and malaria are the three most serious infectious diseases in the world, causing high morbidity and mortality rates especially in low-income and middle-income countries.^2^ The Sustainable Development Goals (SDGs) aim to end malaria, TB and HIV as a public health threat by 2030.^3^ Understanding the spatial distribution of these diseases is essential to inform control and prevention strategies. Although there has been a significant reduction in the global burden of these diseases in the past few decades, they all remain in the top 10 causes of mortality in low-income and middle-income countries.^1^ According to 2020 WHO reports, there was a total of 277 million cases of HIV, TB and malaria and 2.5 million deaths associated with these three diseases globally.^4^ The African continent accounts for a disproportionately high global burden of HIV (73%), TB (25%) and malaria (94%).^4^ While there is considerable geographical overlap in the distribution of these three diseases at regional levels, the codistribution of these diseases is yet to be investigated subnationally in high-burden countries.

The synergy between HIV, TB and malaria infection is strong at an individual level. While TB is the most common opportunistic infection leading to death among people living with HIV,^5^ HIV infection is the most important risk factor for developing active TB.^6^ Studies have also reported that HIV-infected individuals are at increased risk of complicated and severe malaria and death.^7 8^ Malaria and TB are strongly influenced by socioeconomic factors such as housing quality and sanitation.^9^ Previous research has studied the interactions between HIV and TB or malaria,^9 10^ but limited research has investigated the codistribution of all three diseases.

An understanding of the spatial codistribution of these diseases is critical to designing targeted and integrated interventions for surveillance, diagnosis, treatment and prevention that will help achieve the goals of national disease control programmes. Integrated disease control programmes can present cost-effective benefits and synergistic effects compared with vertical programmes.^11 12^ However, accurate knowledge of where to strengthen integrated programmes is key to achieving maximum impact, especially in low-income countries like Ethiopia.

Ethiopia is one of the countries highly affected by HIV, TB and malaria. There are several studies investigating the spatial distribution of HIV, TB and malaria in Ethiopia, which have confirmed the presence of spatial clustering associated with common risk factors such as behavioural, climatic and clinical factors.^13–15^ To the best of our knowledge, this is the first study to combine all three diseases simultaneously to investigate their spatial codistribution. The aim of this study was to develop predictive maps for each of the three diseases, investigate their spatial codistribution, and identify the demographic and climatic factors that influence their distribution in Ethiopia.

## Methods

### Country context

Ethiopia is the second-most populous country in Africa, with an estimated population size of more than 115 million people in 2020.^16^ There are marked differences in population structure, socioeconomic conditions, disease burden and climatic conditions across the country. Ethiopia has a surface area of approximately 1.1 million km² and a population density of 215 people per square kilometre.^16^ It has a variety of geographical features with altitudes ranging from 125 m below sea level to 4620 m above sea level. Ethiopia is administratively divided into ten regional states and two administrative cities (first-level), which are further divided into zones (second-level), districts (third-level), and villages (fourth-level).

In Ethiopia, infectious diseases such as HIV, TB and malaria are the leading causes of morbidity and mortality.^17^ In 2019, it was estimated that there were ~15 000 deaths caused by HIV/AIDS, ~21 000 deaths caused by TB and ~5000 deaths caused by malaria, giving >40 000 deaths caused by the Big Three infectious diseases in Ethiopia.^4 18 19^

The healthcare system of the country contains a mixture of public, private and non-governmental sectors. The public healthcare system is structured into a three-tier system: (1) primary care: composed of health posts, health centres and primary hospitals; (2) secondary care: composed of general hospitals and (3) tertiary care: composed of specialised hospitals.^20^ It is estimated that more than half of the population lives more than 10 km from the nearest health facility, concentrated in regions with poor transport infrastructure.^21^

### Data sources

Data for the primary outcome measures (ie, HIV, TB and malaria prevalence) and exposure variables (ie, climatic variables and population density) were assembled from multiple sources.

HIV prevalence data were obtained from the Ethiopian Demographic and Health Survey (EDHS 2016). The EDHS survey was conducted between January and June 2016 to provide estimates of HIV prevalence based on a nationally representative sample. A finger-prick blood specimen collected from both women and men aged 15–49 years, was tested using an ELISA. All samples testing positive on the first test were subjected to a second test. If the results of the first and second tests were discordant, a third confirmatory assay was used.

TB prevalence data were obtained from the Ethiopian national TB prevalence survey. A detailed description of the survey is provided elsewhere.^22^ Briefly, it was the first nationally representative TB survey conducted in Ethiopia. The survey was conducted between 2010 and 2011, with 85 clusters included in the survey, including 14 clusters in urban areas, 63 clusters in rural areas and 8 clusters in pastoralist areas. Symptom screening, chest X-ray, sputum smear microscopy and TB culture were reported among 46 697 adults and adolescents aged 15 years and above.^23^

Malaria prevalence data were obtained from the Ethiopia national malaria indicator survey, a nationally representative household malaria survey collected between September and December 2015.^24^ Malaria parasite testing was done using multi-species CareStart rapid diagnostic tests and microscopic examination of both thick and thin smear blood slides.^24^ Microscopy slide testing was used for the determination of the prevalence of malaria. The surveys were aggregated to cluster level and malaria prevalence at each cluster was calculated from the number of people who received a diagnostic test and the number of people who tested positive.

Potential covariates were selected based on the availability of country-wide representative data at a high level of resolution and based on biological plausibility and social pathways affecting disease risk and based on them having been previously found to explain spatial variation in risk. Climatic variables such as mean annual temperature and mean annual precipitation were obtained from the WorldClim website.^25^ Altitude data were obtained from the Shuttle Radar Topography Mission.^26^ Data on travel time to the nearest city and travel times to the nearest healthcare facility in minutes (ie, hospital or clinic) were obtained from the Malaria Atlas Project.^27^ Population density, estimated as the number of people per grid cell, was obtained from WorldPop.^28^ Distance to the nearest water body was obtained from previous studies.^29 30^ All these data were extracted at a spatial resolution of 1 km^2^. The data sources of the covariates with their definitions are provided in online supplemental table S1. A polygon shapefile for the Ethiopian administrative boundaries was obtained from the Database for Global Administrative Areas, a free online database.^31^ The dependant variables (HIV, TB and malaria prevalence) were geo-referenced, and covariates were linked to disease prevalence data by extracting their value in the 1 km^2^ grid cell in which each disease prevalence observation was located using ArcGIS (ESRI, Redlands, California, USA) geographical information system (GIS) software.

10.1136/bmjgh-2021-007599.supp1 Supplementary data

### Spatial analysis

Bayesian model-based geostatistics (MBG) was used to generate spatially continuous estimates of the national prevalence of HIV, TB and malaria mapped at a resolution of 1 km^2^. Within the MBG framework, a logistic regression model was fitted to the prevalence data using both fixed effects and spatial random effects. Three different models were constructed independently for the prevalence of HIV, TB and malaria. Here, we present how the model for the prevalence of TB was constructed, but the approach was identical for the other diseases. A spatial binomial regression model was fitted for TB prevalence survey data, including fixed effects for mean annual temperature, mean annual precipitation, altitude, travel time to the nearest city, distance to a water body, population density and geostatistical random effects.^32^ The proportion of TB cases at each surveyed location *j* as the outcome variable was assumed to follow a binomial distribution:

$$Y_{j}∼Binomial\left(n_{j},p_{j}\right)$$

where $Y_{j}$ is the observed number tested positive for TB, $n_{j}$ is the total number of individuals tested for TB and $p_{j}$ is the predicted TB prevalence at location j (*j*=1, …, 85). Mean predicted TB prevalence was modelled via a logit link function with a linear predictor, defined as:

$$logit\left(p_{j}\right)=\alpha +\sum_{z=1}^{z}\beta_{z}X_{z,j}+\zeta_{j}$$

where *α* is the intercept, *β* is a matrix of covariate coefficients, X is a design matrix of z covariates and $\zeta_{j}$ are spatial random effects modelled using a zero-mean Gaussian Markov random field with a Matérn covariance function. The covariance function was defined by two parameters: the spatial scale ρ, which represents the distance beyond which correlation becomes negligible, and σ, which is the marginal SD.^33 34^ Non-informative priors were used for *α* (uniform prior with bounds –∞ and ∞) and we set normal priors with mean=0 and precision (the inverse of variance)=1×10^−4^ for each *β*. We used default priors for the parameters of the spatial random field.^35^ Parameter estimation was done using the Integrated Nested Laplace Approximation (INLA) approach in the R statistical software (R-INLA).^33 34^ Sufficient values (ie, 150 000 samples) from each simulation run for the variables of interest were stored to ensure full characterisation of the posterior distributions.

Predictions of the prevalence of each infection at unsampled locations were made at 1 km² resolution by interpolating the spatial random effects and adding them to the sum of the products of the coefficients for the spatially variant fixed effects at each prediction location.^36^ The intercept was added, and the overall sum was back-transformed from the logit scale to the prevalence scale, providing prediction surfaces that show the estimated prevalence of disease for all prediction locations. An area of coinfection is defined as a geographical area with disease prevalences higher than the upper quartile of 75%. To obtain a co-endemicity map, the spatial predicted prevalence surface for each disease were overlaid in the GIS software. This process allows for the identification of overlapped areas where the prevalence of two or three diseases are highest. This approach has been applied in various studies addressing similar objectives.^37 38^

### Model validation

Models were validated using the conditional predictive ordinates (CPO) and the probability integral transform (PIT) statistics.^39 40^ Both CPO and PIT were obtained as ‘leave-one-out’ cross-validation in INLA. These were defined as follows:${CPO}_{i}=\pi \left(\frac{y_{i}}{y-i}\right)$

$${PIT}_{i}=\pi \left(y_{i}^{new}-\left(y_{i}/y-i\right)\right)$$

The CPO expresses the posterior probability of observing the value of the outcome at location i when the model is fitted to all data except the $y_{i}$. Large values indicate a better fit and small values indicate a poorer fit of the model to that observation and, perhaps, that it is an outlier. PIT measures the probability of a new value $y_{i}^{new}$ to be lower than the actual observed value. For a well-calibrated model, the PIT values should be uniformly distributed. Larger values of CPO and PIT imply a better fit. Models with different combinations of covariates were constructed and compared. The Watanabe-Akaike Applicable Information Criterion (WAIC) statistic was used to select the best-fitting model.

## Results

**Figure 1:**
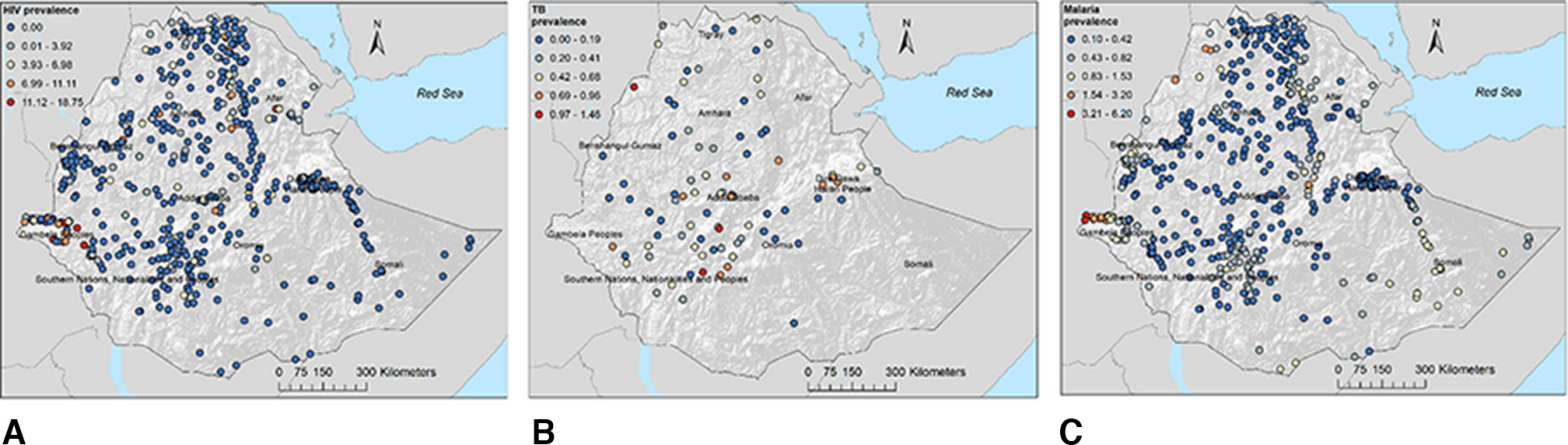
Geographical locations of data points and prevalence of (A) HIV, (B) tuberculosis and (C) malaria in Ethiopia.

Data were available from 643 georeferenced locations for HIV, 85 for TB, and 643 for malaria. The survey locations covered all regions of the country (figure 1).

### Prevalence of HIV, TB and malaria at national and regional levels

Table 1 shows the national and regional prevalence of HIV, TB and malaria in Ethiopia. The national prevalence was 1.54% for HIV (95% CI 1.40 to 1.70), 0.39% (95% CI 0.34 to 0.45) for TB and 1.1% (95%CI 0.95 to 1.32) for malaria among all ages. Substantial variation was observed in the prevalence of these diseases at regional levels, with the highest prevalence of TB observed in Dire Dawa (0.96%) and Gambela (0.88%) regions and the highest prevalence of malaria observed in Gambela (6.1%) and Benishangul-Gumuz (3.8%) regions. The prevalence of HIV was highest in Gambela (4.52), Addis Ababa (3.52%) and Dire Dawa (2.67%) regions and lowest in Somali (0.09%), Southern Nations, Nationalities, and People’s (0.36%) and Benishangul-Gumuz regions (0.79%).

**Table 1:** National and regional prevalence of TB, HIV and malaria in Ethiopia

Regions	HIV prevalence (%)	TB prevalence (%)	Malaria prevalence (%)
Addis Ababa	3.52	0.67	NA
Afar	1.29	0.57	0.3
Amhara	1.19	0.32	0.8
Benishangul-Gumuz	0.79	0.00	3.8
Dire Dawa	2.67	0.96	0.0
Gambela	4.52	0.88	6.1
Oromiya	1.21	0.33	0.3
SNNPR	0.36	0.50	0.5
Somali	0.09	0.43	0.0
Tigray	1.08	0.30	0.8
**Ethiopia**	1.52	0.39	1.1

HIV, Human immunodeficiency virus; NA, Not avialable; SNNPR, Southern Nations, Nationalities, and People’s Region; TB, tuberculosis.

### Spatial distribution of HIV, TB and malaria prevalence

**Figure 2:**
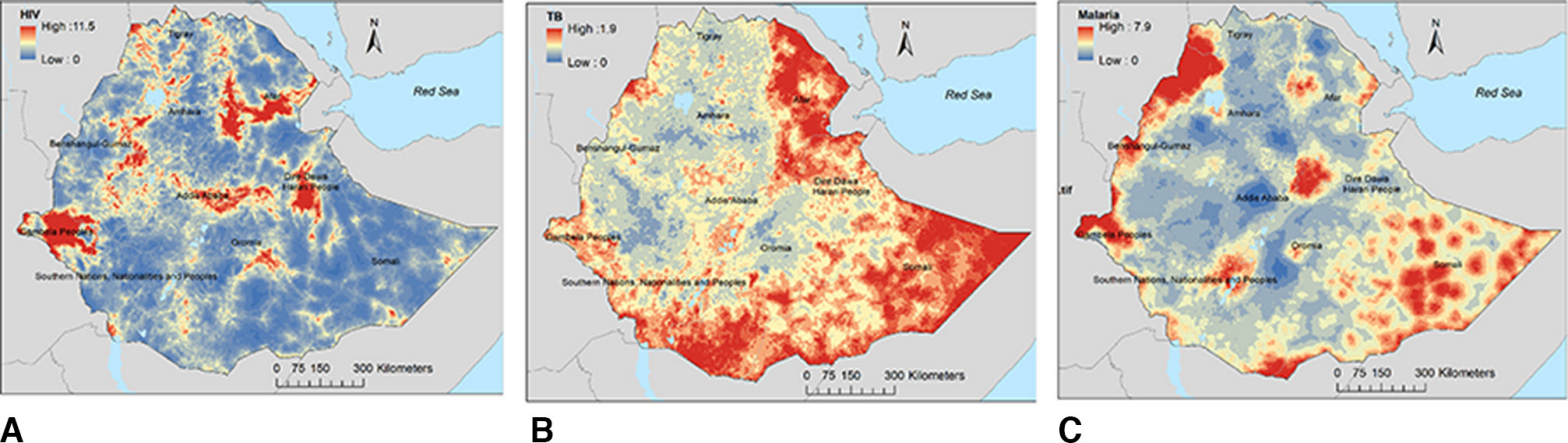
The predicted geospatial maps for the prevalence of (A) HIV, (B) tuberculosis and (C) malaria in Ethiopia.

The prevalence of HIV, TB and malaria varied substantially within regions. Figure 2 shows the predicted prevalence of HIV, TB and malaria in Ethiopia at the pixel level. The prevalence of HIV was spatially varied, with the highest prevalence (ie, hotspot areas) predicted in Gambela region and major cities such as Addis Ababa, Dire Dawa, Harer and Desie (figure 2A). The peripheral areas of the country (eg, Afar and Somali regions) bordering Djibouti, Somalia, Eritrea and Kenya had the highest prevalence of TB while the central, northern and western parts of the country had the lowest prevalence of TB (figure 2B). High malaria prevalence was predicted in the northwest (eg, Humera, Metema, Sanja, Quara) and eastern (eg, Kebridehar, Gode) parts of the country and in the Great Rift Valley (figure 2C). In contrast, a low prevalence of malaria was predicted in the central parts of the country. Prediction uncertainty, as indicated by a high SD, was greatest in the border regions (Afar and Somali) for all diseases (online supplemental figure S1).

### Spatial codistribution of HIV, TB and malaria prevalence

**Figure 3:**
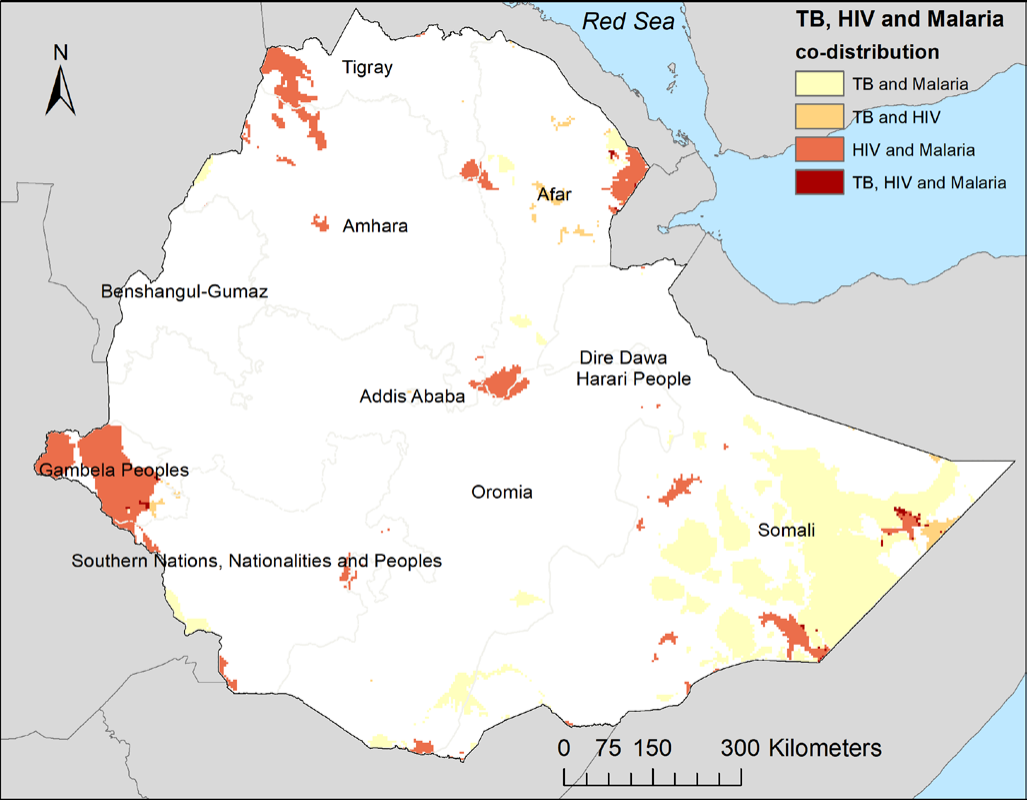
Predicted areas of codistribution for tuberculosis, HIV and malaria, Ethiopia. High prevalence is defined as a prevalence of more than the upper quartile.

Areas of spatial overlap of combinations of two or three diseases were predicted in focal areas across the country (figure 3). For example, the burden of all three diseases was high in Gambela region. Geographical overlap of high TB and HIV prevalence was also observed in the Afar region.

### Drivers of HIV, TB and malaria prevalence

Table 2 shows the results of the Bayesian geostatistical models. Travel times to the nearest city in minutes (mean regression coefficient (*β*): –0.532; 95% credible interval (95% CrI) –0.960 to –0.122) was negatively associated with HIV prevalence; whereas population density (people per square kilometre, *β*: 0.010; 95% CrI 0.005 to 0.014) and distance to water body (km, *β*: 0.182; 95% CrI 0.053 to 0.311) were positively associated with HIV prevalence. Population density was also positively associated with TB prevalence (*β*: 0.008; 95% CrI 0.001 to 0.014). Climatic factors such as annual mean temperature (°C, *β*: 0.346; 95% CrI 0.258 to 0.434) and annual mean precipitation (mm, *β*: 0.312; 95% CrI 0.262 to 0.362) as well as travel time to the nearest city in minutes (minutes, *β*: 0.113; 95% CrI 0.084 to 0.142) were found to be positively associated with malaria prevalence. In contrast, population density (*β*: –0.005; 95% CrI –0.006 to –0.004) and distance to health facility (minutes, *β*: –0.300; 95% CrI –0.347 to –0.254) were negatively associated with malaria prevalence.

**Table 2:** Regression coefficient mean and 95% credible intervals (CrI) of covariates included in a Bayesian spatial model with Binomial response for the prevalence of tuberculosis, HIV and malaria in Ethiopia

Covariates	HIV	Tuberculosis	Malaria
	Regression coefficients Mean (95% CrI)	Regression coefficients Mean (95% CrI)	Regression coefficients Mean (95% CrI)
Temperature	0.23 (−0.92 to 1.430)	−0.62 (−1.58 to 0.35)	**0.34 (0.26 to 0.43**)
Precipitation	−0.01 (−0.47 to 0.426)	−0.22 (−0.57 to 0.09)	**0.31 (0.26 to 0.36**)
Altitude	−0.11 (−1.31 to 1.133)	−0.72 (−1.76 to 0.33)	−0.06 (−0.15 to 0.02)
Travel time to city	−**0.53 (−0.96 to –0.122**)	0.18 (−0.12 to 0.48)	**0.11 (0.08 to 0.14**)
Population density	**0.01 (0.005 to 0.014**)	**0.008 (0.001 to 0.014**)	−**0.005 (−0.006 to –0.005**)
Distance to water body	**0.18 (0.05 to 0.31**)	0.05 (−0.13 to 0.24)	−0.002 (−0.011 to 0.007)
Distance to health facility	−0.44 (−1.04 to 0.12)	−0.29 (−0.91 to 0.29)	−**0.300 (−0.35 to –0.254**)
Intercept	−5.97 (−6.67 to –5.37)	−5.58 (−6.42 to –4.90)	−5.667 (−5.88 to –5.44)

Bold shows ‘statistically significant’ results within a Bayesian framework (no zero within the 95% CrI).

Results of model validations for HIV prevalence (online supplemental figure S2), TB prevalence (online supplemental figure S3) and malaria prevalence (online supplemental figure S4) are presented in online supplemental file 1. The CPO and PIT indicated that the predictive models were well fitted. According to the WAIC statistic, the model that contained all covariates was the best-fitting model for all diseases (online supplemental table S2).

## Discussion

The national HIV prevalence in Ethiopia was 1.5%, which is lower than the African HIV prevalence (3.9%) but nearly two times the global average HIV prevalence (0.8%).^41^ The prevalence of TB in Ethiopia was 0.39%, which is similar to other African countries such as Kenya (0.56%),^42^ Zambia (0.63%)^43^ and Gambia (0.21%),^44^ but higher than other high TB burden countries in Asia such as India (0.03%)^45^ and China (0.06%).^46^ The prevalence of malaria in our study was 1.1%, which is lower than in other African countries, but it varied greatly at a lower administrative level,^47^ with large populations still exposed to substantial malaria risk.

Our study showed that remoteness, demography and climatic factors were associated with the spatial distribution of HIV, TB and malaria. As the transmission mechanisms and preventive measures of TB, HIV and malaria are complex and multi-factorial, there are some risk factors that affect the spatial codistribution of the three diseases. For example, our study showed that population density was a common variable in all three diseases which was positively associated with both HIV and TB prevalence and negatively associated with malaria prevalence. Our study also showed a positive association of distance to a water body with HIV prevalence while a negative effect on travel times and a positive effect on population density was observed. Staying further away from water bodies may be a proxy indicator of economic, environmental and social needs which may have an impact on HIV prevalence. For example, food insecurity which can be caused by a lack of water sources can drive sexual risk-taking behaviours and migration, as well as increase susceptibility to infections that are common among people living with HIV.^48^ Moreover, longer travel times to cities may indicate low urbanicity and a low population density which favours a lower risk of HIV and TB coinfection. Previous studies also found that people living in a capital city were at a high risk of TB and HIV infection.^13 49^ This may be because transmission of TB and HIV may be more common in urban settings due to overcrowding and higher population density. Other explanations aside from the close association between HIV and TB may be indirect factors related to low income, high rates of migration especially in infected individuals migrating from high prevalent areas, as well as high levels of social networking. HIV risk behaviours such as commercial sex work and drug use are also common in capital cities.^50^ Consistent with previous studies, climatic factors such as high annual mean temperature and high annual mean precipitation as well as long travel time to the nearest city were positively associated with malaria prevalence.^51 52^ In contrast, the population density was negatively associated with malaria prevalence, which is not surprising given malaria is more common in rural areas.

Substantial spatial variation was observed in all three diseases at regional and local levels in Ethiopia. Previous studies have reported similar spatial clustering of HIV, TB and malaria in Ethiopia.^13 15 53^ However, the current study provided additional information in which the spatial distribution of HIV, TB and malaria overlapped in some parts of the country. For example, hotspots of a high prevalence of all three diseases were observed in Gambela region. This region is located in the west of Ethiopia, bordering South Sudan, and characterised by low healthcare access, low socioeconomic index and high temperature and rainfall.^54^ These demographic and climatic factors have been reported as some of the main drivers of TB transmission.^55^ The high prevalence of HIV, TB and malaria along the border areas might be due to inadequate case management and weaker healthcare systems.^54^ It could be also due to cross-border travel and high rates of infection across the border.^56^ Previous studies in Ethiopia showed that malaria transmission is endemic in lowland areas with warm and humid climates like the Gambela region and appears to be epidemic in highland areas.^57^ The high prevalence of HIV in Gambela region could be due to cultural practices such as polygamy practices and male uncircumcision.

Spatial overlap of TB and HIV prevalence was also observed in Harari, Dire Dewa and Afar regions. While there were hotspots of TB and malaria in the Somali region, there was little HIV in this region. In addition, while TB and malaria hotspots were generally observed in the most rural and peripheral areas sharing international borders, high HIV prevalence was mostly observed in the capital cities. These findings suggested that although there was overlap in the distribution of infectious diseases in some parts of the country, this was not the case throughout the country. This highlights that targeting service integration approaches that consider the profile of diseases at a local level would be more effective than nationwide service integration. Geographically targeted service integration may enhance the efficiency and cost-effectiveness of disease control programmes. Thus, mapping the codistribution of infectious diseases such as HIV, TB and malaria would be a key step in strengthening integrated disease control programmes.

The SDGs articulate a goal to end HIV, TB and malaria epidemics by 2030.^3^ Health service integration has been recommended by WHO as one strategy to enhance the prevention and control of these diseases. Integration of TB, HIV and malaria services has been implemented in many resource-limited countries including Ethiopia.^58^ Ethiopia has implemented an Integrated Disease Surveillance and Response (IDSR) strategy since 1996,^59^ which has made a significant contribution to the control and prevention of communicable diseases by filling the gaps observed in vertical disease control programmes.^60^ However, several challenges were identified with the implementation of IDSR such as limited financial resources, lack of coordination, inadequate training and supervision.^60^ Targeting the IDSR strategy according to local disease profiles may help overcome these challenges.

This study has some important limitations, including the difference in data collection periods. While the data for HIV and malaria were collected in 2016 and 2015, respectively, the data for TB were collected between 2010 and 2011. Additionally, due to a lack of available data, some important ecological level variables were not included in our geospatial models, which might affect the validity of the prediction maps. Finally, the data on TB was much sparser than for malaria and HIV, and the spatial predictions are therefore likely to be less robust and strongly driven by the effect of covariates particularly in areas with no observed data on those which are sparsely populated. In another study we have investigated supplementing the national survey on TB with data from other studies, using a geospatial meta-analytic approach.^22^

## Conclusion

Our study found that the national prevalence of TB, HIV and malaria varied substantially at subnational and local levels. The spatial distribution of the diseases was associated with demographic and climatic factors. Spatial overlap of TB, HIV and malaria prevalence was observed in some parts of the country, with one area with a high prevalence of all three diseases being the Gambela region. This highlights that targeting service integration approaches at a local level would be more effective than nationwide service integration. These findings can guide policymakers in Ethiopia to design geographically targeted and integrated disease control programmes to achieve maximum impact.

## Data Availability

All data relevant to the study are included in the article or uploaded as supplementary information.
