# Supplementary material for: Spatial codistribution of HIV, tuberculosis and malaria in Ethiopia
Source: BMJ Glob Health. 2022 Feb 22;7(2):e007599. doi: 10.1136/bmjgh-2021-007599 (PMC8867247; doi:10.1136/bmjgh-2021-007599)
Supplement: Supplementary data [file bmjgh-2021-007599supp001.pdf]

Supplementary information

Spatial co-distribution of HIV, tuberculosis, and malaria in Ethiopia

Kefyalew Addis Alene[1,2], Ahmed Elagali[1], Dylan D. Barth[1,3], Susan F Rumisha[1,4], Punam Amratia[1], Daniel J Weiss[1,2], Kendalem Asmare Atalell[5], Andargachew Kumsa[6], Peter W Gething[1,2], Archie C A Clements[1,2]*

[1]Telethon Kids Institute, Nedlands, Western Australia, Australia
[2]Faculty of Health Sciences, Curtin University, Bentley, Western Australia, Australia
[3]The University of Western Australia, Perth, Western Australia, Australia
[4]National Institute for Medical Research, Headquarters, Dar es Salaam, Tanzania
[5]College of Medicine and Health Sciences, University of Gondar, Gondar, Amhara, Ethiopia
[6]Ethiopia Ministry of Health, National TB Control Program, Addis Ababa, Ethiopia

Alene KA, *et al. BMJ Global Health* 2022; 7:e007599. doi: 10.1136/bmjgh-2021-007599

Supplementary tables

Table S1: Data sources and definitions of covariates

Covariates	Data sources	Definitions
Population density	WorldPop	Number of people per square kilometre (grid) [1]
Travel times to cities	Malaria Atlas Project (MAP)	Travel time in minutes to the nearest city with a population of more than 50,000 [2]
Temperature	WorldClime	Annual mean environmental air temperature (°C) [3]
Precipitation	WorldClime	Annual mean rainfall (mm) [3]
Altitude	Shuttle Radar Topography Mission (SRTM)	Elevation of the earth land surface in km [4]
Distance to water body	Global Lakes and Wetlands Database (GLWD)	Distance to permanent and semi-permanent water based on presence of lakes, wetlands, rivers and streams, and accounting for slope and precipitation [5]
Access to healthcare facilities	Malaria Atlas Project (MAP)	Walking travel times in minutes to the nearest health facility [6]

Table S2: Watanabe-Akaike information criterion (WAIC) values corresponding to different model specifications.

Model specifications	WAIC		
	HIV	TB	Malaria
Temperature	1319.9	311.7	15727.0
Temperature + Precipitation	1320.7	313.6	15518.7
Temperature + Precipitation + Altitude	1322.4	314.1	15548.6
Temperature + Precipitation + Altitude + Travel time to city	1314.8	313.1	15532.4
Temperature + Precipitation + Altitude + Travel time to city + Population density	1305.4	315.4	15357.0
Temperature + Precipitation + Altitude + Travel time to city + Population density + distance to water body	1299.2	317.3	15396.1
Temperature + Precipitation + Altitude + Travel time to city + Population density + Distance to water body + Access to health care	1291.3	308.2	15212.2

Alene KA, *et al. BMJ Global Health* 2022; 7:e007599. doi: 10.1136/bmjgh-2021-007599

Supplementary figures

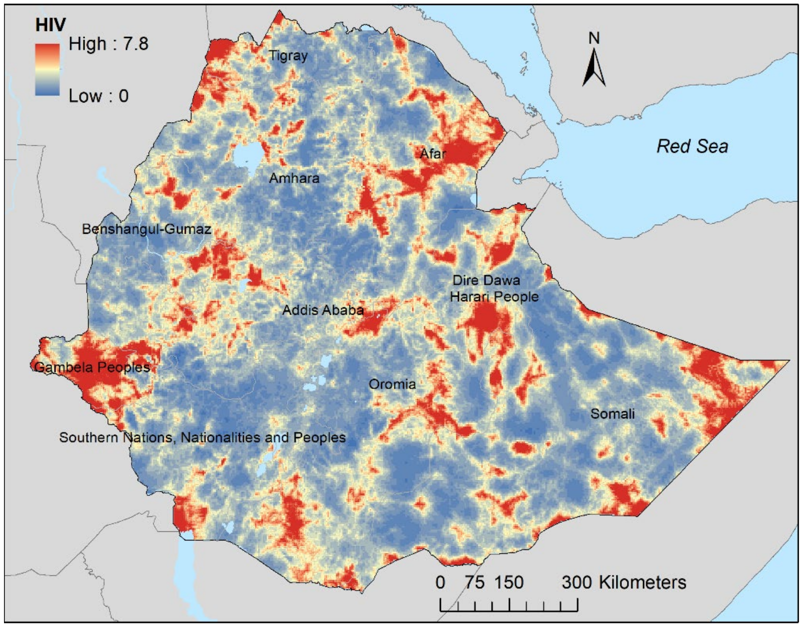

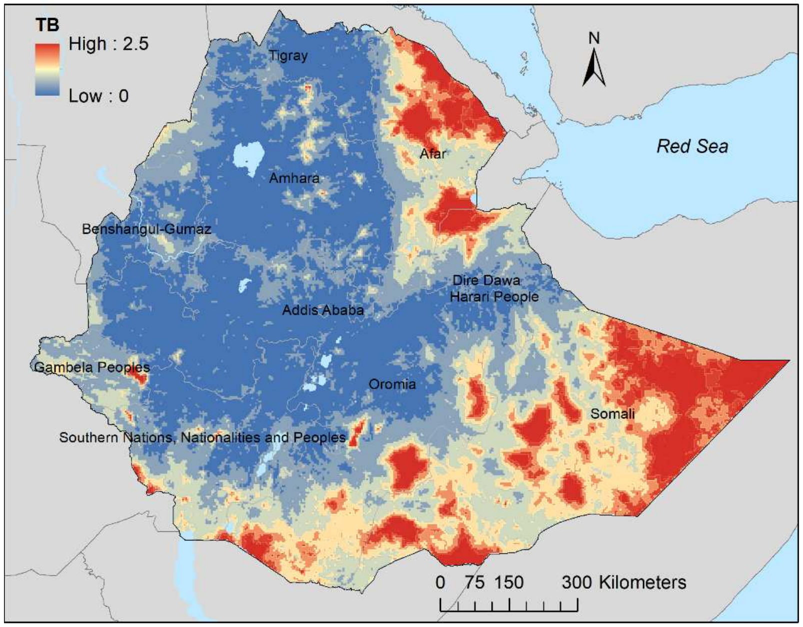
TB
High : 2.5
Low : 0
N
Red Sea
Tigray
Afar
Amhara
Benshangul-Gumaz
Dire Dawa
Harari People
Addis Ababa
Gambela Peoples
Oromia
Somali
Southern Nations, Nationalities and Peoples
0 75 150 300 Kilometers

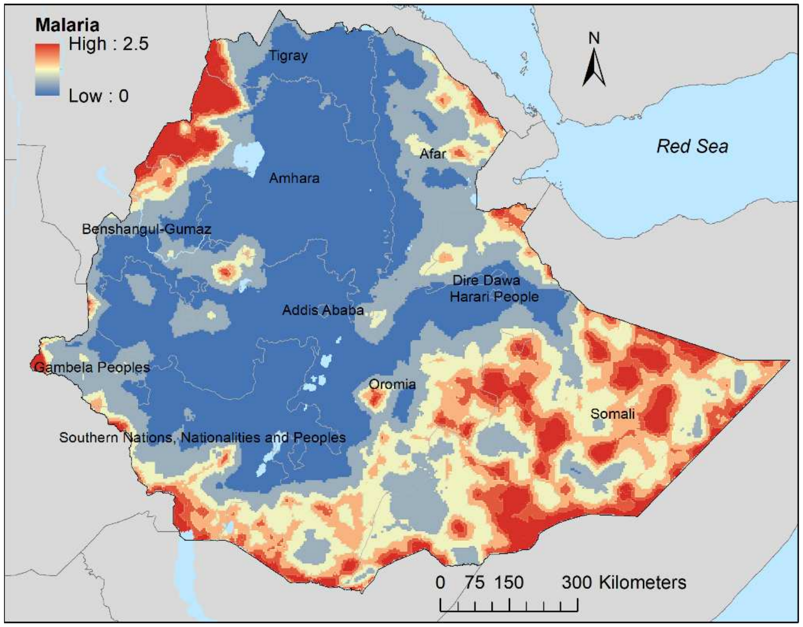

Alene KA, *et al. BMJ Global Health* 2022; 7:e007599. doi: 10.1136/bmjgh-2021-007599

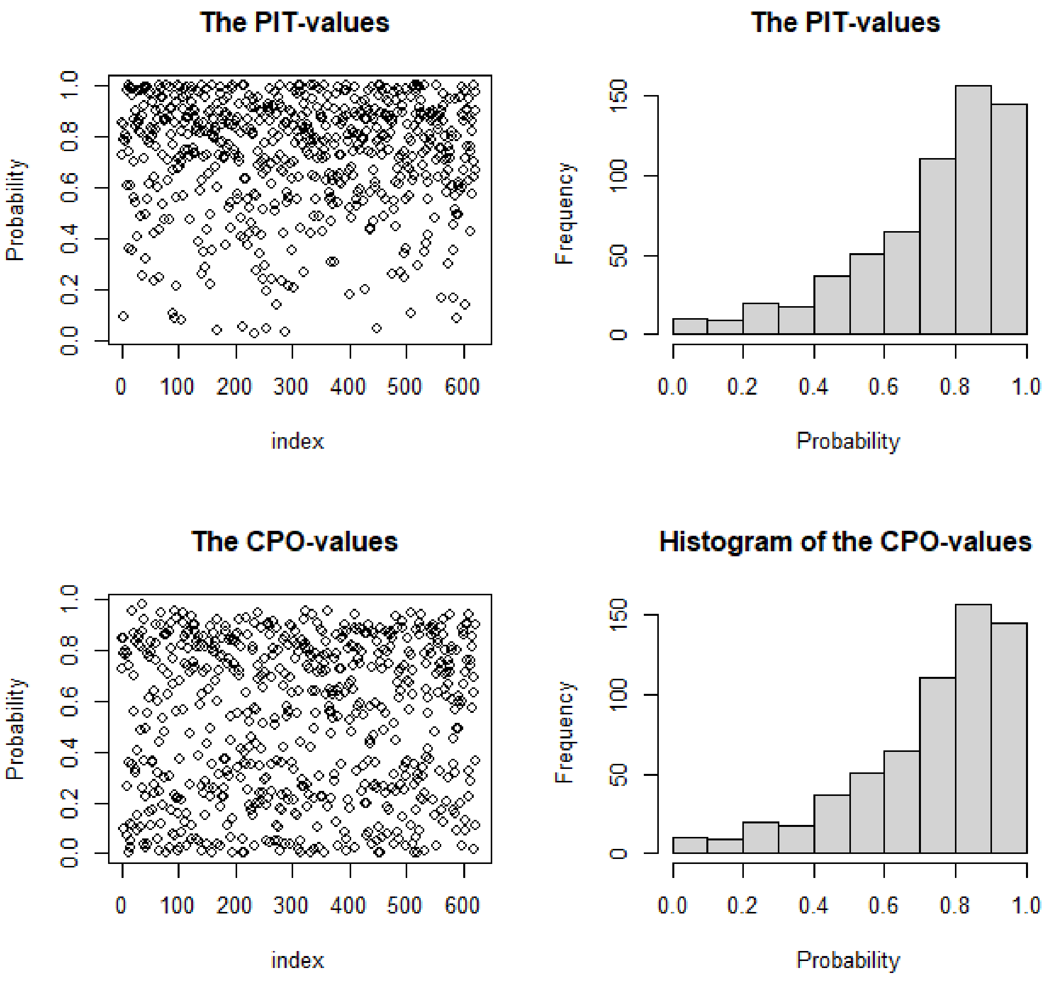

Alene KA, *et al. BMJ Global Health* 2022; 7:e007599. doi: 10.1136/bmjgh-2021-007599

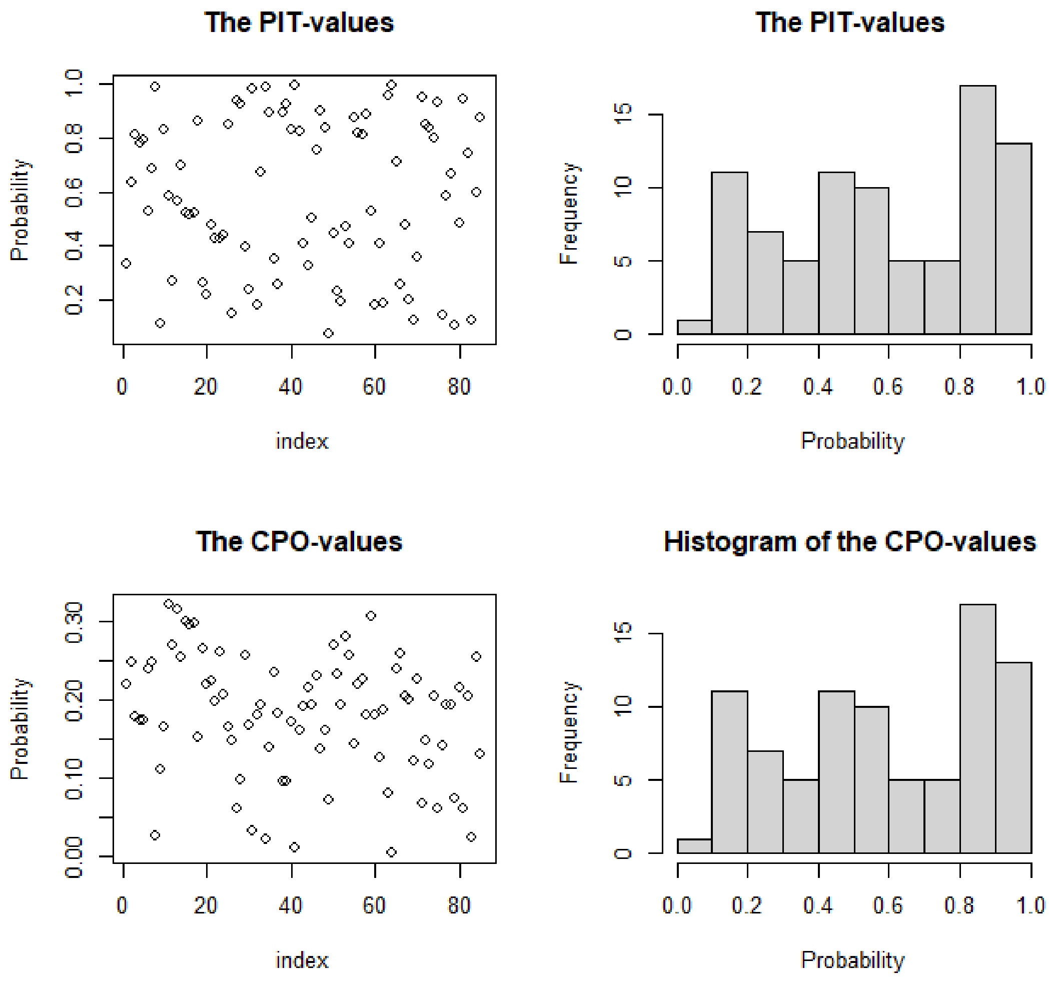

Alene KA, *et al. BMJ Global Health* 2022; 7:e007599. doi: 10.1136/bmjgh-2021-007599

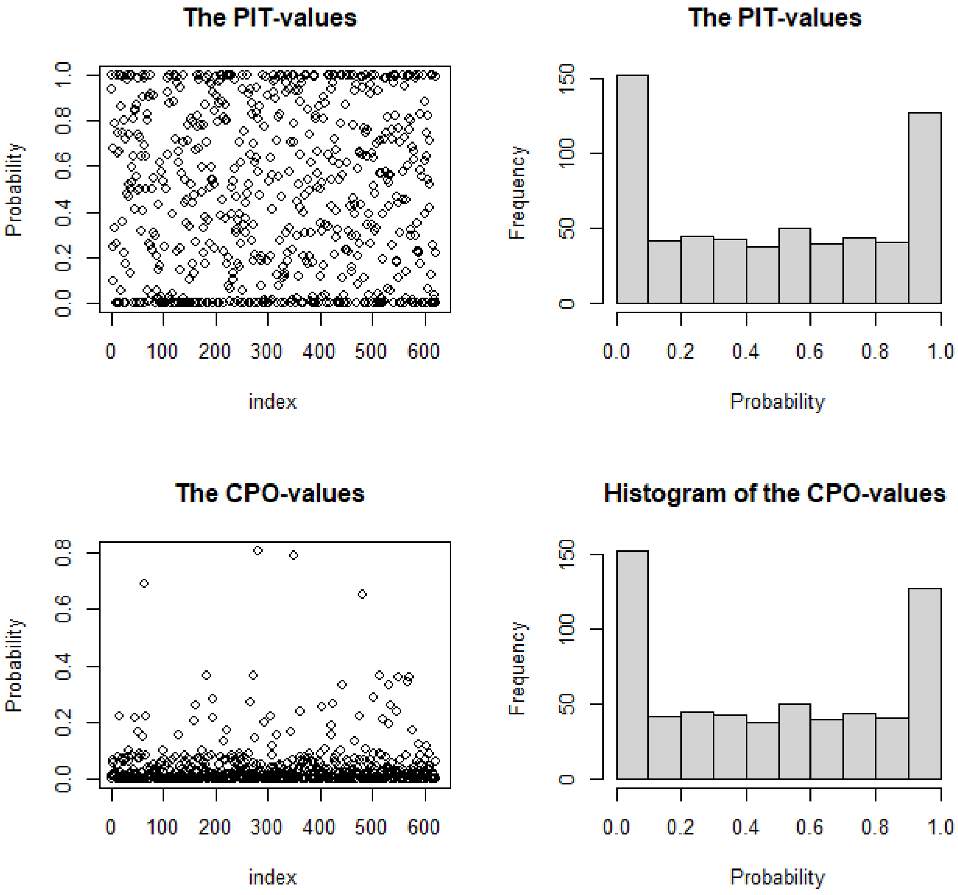
The PIT-values
Probability
index
0.0
0.2
0.4
0.6
0.8
1.0
0
100
200
300
400
500
600
The PIT-values
Frequency
Probability
0
50
100
150
The CPO-values
Probability
index
Histogram of the CPO-values
Frequency
Probability

References

1. Tatem AJ. WorldPop, open data for spatial demography. *Scientific data* 2017; **4**(1): 1-4.
2. Weiss DJ, Nelson A, Gibson H, et al. A global map of travel time to cities to assess inequalities in accessibility in 2015. *Nature* 2018; **553**(7688): 333-6.
3. Fick SE, Hijmans RJ. WorldClim 2: new 1-km spatial resolution climate surfaces for global land areas. *International journal of climatology* 2017; **37**(12): 4302-15.
4. Van Zyl JJ. The Shuttle Radar Topography Mission (SRTM): a breakthrough in remote sensing of topography. *Acta Astronautica* 2001; **48**(5-12): 559-65.
5. Lehner B, Döll P. Global lakes and wetlands database glwd. *GLWD Docu mentation* 2004.
6. Weiss D, Nelson A, Vargas-Ruiz C, et al. Global maps of travel time to healthcare facilities. *Nature Medicine* 2020; **26**(12): 1835-8.
